# Coconut shells as filling material for anaerobic filters

**DOI:** 10.1186/2193-1801-2-655

**Published:** 2013-12-05

**Authors:** Luana Mattos de Oliveira Cruz, Ronaldo Stefanutti, Bruno Coraucci Filho, Adriano Luiz Tonetti

**Affiliations:** Architecture and Urbanism, FEC/UNICAMP, University of Campinas-School of Civil Engineering, Campinas, SP Brazil; Center for Technology, Federal University of Ceará, UFC, Fortaleza, Brazil; University of Campinas-School of Civil Engineering, Architecture and Urbanism, FEC/UNICAMP, Cidade Universitária “Zeferino Vaz”, Avenida Albert Einstein, 951, PO Box 6021, CEP: 13083-852 Campinas, SP Brazil

**Keywords:** Anaerobic filter, Anaerobic treatment, Coconut, Decentralized sanitation, On site sewage treatment, Sewage

## Abstract

In rural areas of developing countries, there is a lack of sanitation services and the installation of such infrastructure is hampered by the high investment costs for initial implementation and by the limited availability of qualified personnel. An alternative to traditional sanitation services include an anaerobic filter, but the high cost of appropriate filling material can be an obstacle to its wide-spread implementation. To decrease this construction cost, the objective of this work was to study the use of coconut shells as filling material for anaerobic filters. Anaerobic filters were built and filled with the studied material and operated with up flow and hydraulic retention time of 9 hours. The reactors provided a removal of 79 ± 16% in BOD terms, indicating that the coconut shell filling had efficiency consistent with the literature data. In addition, the husks were found to retain their tensile strength following use in the reactors. Coconut husks have more empty bed volume than other low cost materials, such as crushed stone, nearing properties of traditional materials. The results of this study indicate that coconut husks may prove to be a low cost alternative to traditional fillers for anaerobic treatment in rural communities.

## Introduction

It is estimated that over 82% of the rural population in developing countries have poor sanitation services (Alexiou and Mara 2003;Massoud et al. 2009). In Brazil alone, 31 million people live outside major urban centers (IBGE 2010) and a large portion of their waste is discarded in local bodies of water or surface soil, threatening the quality of water used for public supply. In conjunction with this lack of centralized sanitation, residents of rural areas have little capacity to invest in infrastructure and a lack of technical understanding to drive implementation (Paraskevas et al. 2002;Tonetti et al. 2012), hindering the installation of sophisticated and costly systems to treat sewage.

The Brazilian Research Programme on Basic Sanitation (PROSAB) has completed numerous research studies investigating methods to improve sanitation in rural areas. One of the reactors studied was the anaerobic filter, which is a relatively low cost option with a compact design which produces a small amount of excess sludge (Camargo and Nour 2001;Baek et al. 2010;Frankin 2001), making it suitable for decentralized sanitation in rural communities (Hedberg 1999;Wilderer and Schreff 2000).

One of the greatest impediments to the adoption of such treatment on a wide-spread scale is the cost of the filling material (Frankin 2001), which alone can be as costly as the reactor construction (Van Haandel et al. 2006). Therefore, alternative materials have been studied to replace traditional materials such as crushed stone and plastic rings. Low cost materials being evaluated include cut conduit rings, ground granulated blast furnace slag, bamboo rings and ceramic bricks (Camargo and Nour 2001;Tonetti et al. 2011;Chernicharo 2006), glass (Show and Tay 1999), loofah sponge (Yang et al. 2004), porous floating ceramic media (Kang et al. 2003) and ground tire rubber (Barros et al. 2011).

Seeking to increase the possibility of using anaerobic filters in small villages, it was found that coconut shells of the *Cocos nucifera* species could be used as a filler medium. Such coconuts are widely available in many countries, especially in developing countries, such as India, Philippines, Indonesia, Sri Lanka, Thailand, Mexico and Brazil (EMBRAPA 2011). Brazil produces more than 1 billion coconut fruits per year (EMBRAPA 2011) and, as a consequence, there is an enormous amount of waste husks, boosting several research studies investigating the utilization of these shells in new products or processes.

In Brazil, despite the spread of UASB (Upflow Anaerobic Sludge Blanker) reactor (Rosa et al. 2012), in small communities use of anaerobic filters would still be more attractive. The anaerobic filters are more easily constructed, requiring only a tank and filling material. The filling material makes it difficult to washout the sludge and particulate organic matter, ensuring a better quality effluent even with the existence of large fluctuations in flow, characteristic of small treatment systems. The filling material also assists in the proper sewage flow distribution, hampering the formation of short circuits. In comparison, the UASB reactor requires the gas-liquid separator, which requires greater care in its design and construction. Additionally, in such a system, if the sewage distribution system is not built and maintained correctly, there will be the risk of forming preferential flow channels, impairing treatment.

For these reasons, the present study focuses on the use of coconut shells of the *Cocos nucifera* species as a medium for anaerobic filters, evaluating their resistance to biological and chemical degradation due from sewage interaction. Additionally, this study assesses ability of coconut husks to generate effluent that meets legal standards. This makes it possible to evaluate the applicability of an urban waste, the husks, as filling material for anaerobic filters, reducing the cost of construction of the reactor and allowing the recycling of coconut shells.

## Materials and methods

**Figure 1:**
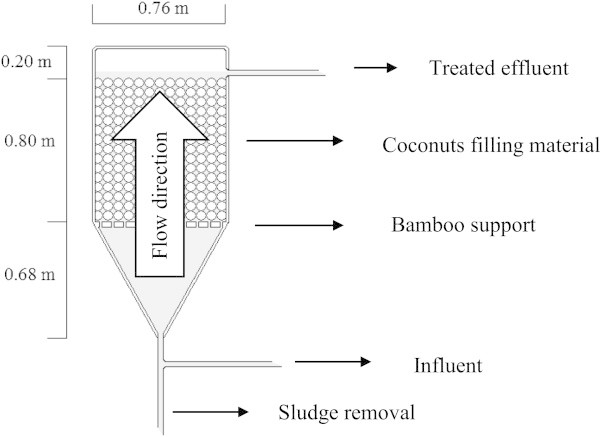
**Scheme of the up flow anaerobic filter (Tonetti et al.** 2012 **).**

This project was carried out in a research area of the School of Civil Engineering, Architecture and Urbanism, University of Campinas (UNICAMP). The raw sewage was collected from the sewage collection network within the university campus, with a portion of its flow directed to four anaerobic filters. The reactors were made of stainless steel with cylindrical shape and total volume of 500 L (Figure 1). The filters were 1.68 m in length and 0.76 m in diameter. The conical bottom was separated from the region occupied by pieces of coconut shells by a bamboo grid (Cruz et al. 2010 and Tonetti et al. 2012). The grid was also used to better distribute the sewage.

**Figure 2:**
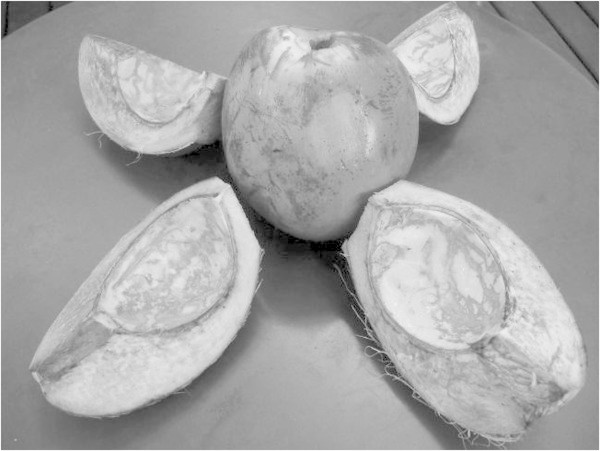
**Coconut shells of the** ***Cocos nucifera*** **species.**

The material used to fill the anaerobic filters was coconut shells of the *Cocos nucifera* species, broken into four pieces as shown in Figure 2. Approximately 280 kg of this material was needed to fill each reactor.

The operation of the anaerobic filters was with up flow and hydraulic retention time (HRT) of 9 hours. This HRT duration was chosen based on previous experiments conducted by Cruz et al. (2010). Cruz et al. (2010) studied this reactor after the starting period and, in this article, we seek to provide a greater amount of data.

Samples of raw sewage and effluent from the four anaerobic filters were obtained weekly, and analyses of pH, total alkalinity, chemical oxygen demand (COD), biochemical oxygen demand (BOD), total suspended solids, volatile suspended solids, phosphate and Kjeldahl nitrogen (TKN) were performed according to *Standard Methods for the Examination of Water and Wastewater*^2012^).

The data presented in this paper was obtained over a two year period. The reactors start–up occurred without inoculation and lasted for three months, until the reactor reached a steady state of various physico-chemical parameters such as chemical oxygen demand (COD), biochemical oxygen demand (BOD).

### Tensile test

Tensile testing of the coconut shells was done using mechanical equipment from MTS (810-TestStarII Model). For each tensile test ten fibers removed from coconut husks were used. The fibers were twisted for the measurement of its diameter with a caliper and were subjected to a load of increasing force until failure. From the data obtained and the sectional area, we found the tensile strength by using Equation 1. The coconut husks samples were extracted portions of shells prior to and after being used as filler material in the reactor.
1

Where *T* is the value of tensile strength (MPa), *F* is the maximum force applied (N) up to its rupture and *A* is the estimated area (mm^2^) from the diameter measured. The speed at which the claws distanced was 0.020 m.min^-1^.

### Density, empty bed volume and surface area

The density of the coconut shells was determined using the principle of mass and water displacement. A 10 L vessel was utilized in which 5 L of water was loaded and the mass and temperature of this set-up was determined. Following this, coconut shells were loaded into the vessel until the maximum volume of the vessel was reached, and the new mass was recorded. From the obtained data of weight and volume displacement of the liquid in the vessel, the density (kg.m^-3^) was determined.

To find the empty bed volume, the same methodology used to obtain the shell density was utilized. The displacement volume of the liquid caused by the introduction of coconut shells on the vessel was used and this was related to the original volume of liquid, according to the Brazilian guidelines (NBR NM45 2006).

To determine the surface area of the material, husk surfaces were coated with paint and the surface was super-positioned on black paper. The picture that appeared on the dark surface was scanned and the area was determined using Autocad. This data is then correlated with the volume area, obtaining the surface area in m^2^.m^-3^.

## Results and discussion

Throughout the research testing period, the average temperature was 25.4 ± 4.5^o^C and the pH values were 7.2 ± 0.3 for the raw sewage sample and 7.2 ± 0.5 for the effluent of the anaerobic filters. Total alkalinity averaged 156 ± 35 mgCaCO_3_L^-1^ and 301 ± 100 mgCaCO_3_L^-1^, respectively for raw sewage and anaerobic effluent. The increased alkalinity observed for the anaerobic effluent indicated that the coconut shells do not impair the buffering capacity of the system.

**Figure 3:**
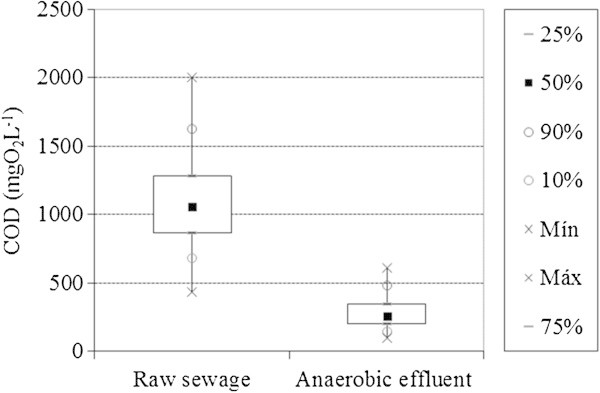
**Average COD of the raw sewage and effluent from anaerobic filters.**

As for chemical oxygen demand (COD), the mean found for the raw sewage was 1105 ± 338 mgO_2_L^-1^ (Figure 3). This figure is above the range considered as typical by Metcalf and Eddy (2003). This is attributed to the sewage collection area utilized, which is believed to receive an abnormally high concentration of organic matter as it was inserted on a University campus.

After the anaerobic treatment, the effluent reached a COD of 281 ± 118 mgO_2_L^-1^, indicating a removal of 73 ± 12% of the organic matter in COD terms. This removal is similar to averages from 65 to 80% for other anaerobic reactors (Foresti 2002) and is consistent with the efficiency found by other authors such as Cruz et al. (2010) and Camargo and Nour (2001), who used bamboo as a medium for anaerobic filters and who obtained removals in the range from 60 to 80%.

Despite the high percentage of removal of organic matter, the effluent still possessed COD values above the maximum allowable limit for release into bodies of water appointed by the laws of the Brazilian State of Minas Gerais (COPAM 2008), which is 180 mgO_2_L^-1^. This is one of the few Brazilian standards that indicate the maximum COD value acceptable to discharge into the environment. From this information, it appears that anaerobic filters, irrespective of the material employed as filling, require a post-treatment to achieve COD levels below the specified maximum value.

**Figure 4:**
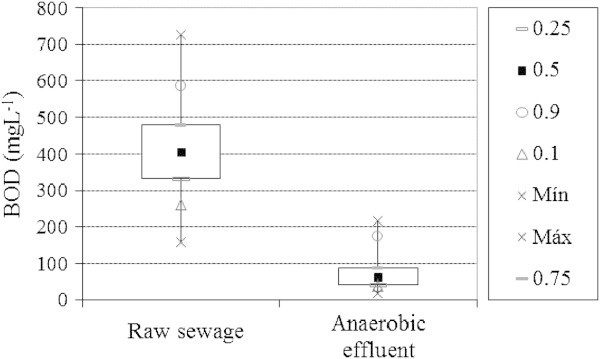
**Average BOD of the raw sewage and effluent from anaerobic filters.**

The biochemical oxygen demand (BOD) for raw sewage averaged 419 ± 79 mgL^-1^ (Figure 4). The anaerobic effluent reached 79 ± 54 mgL^-1^, providing an efficiency of 79 ± 16% over the raw sewage. This removal obtained can also be compared to the survey conducted in Brazil by Cruz et al. (2010) and Pinto (1995). The researchers found that, for a series of surveys which used anaerobic filters operated with hydraulic retention times ranging between 6 and 8 hours, average efficiencies ranged between 68 and 79%. Thus, it is clear that the reactors studied in this work achieved results comparable to the previous studies.

The removal of nitrogen and phosphorus was negligible, which is expected when comparing similar previous studies (Mergaert et al. 1992). The raw sewage had a Kjeldahl nitrogen (TKN) concentration of 58 ± 13 mgL^-1^ and the anaerobic effluent had a concentration of 52 ± 9 mgL^-1^, with no significant difference between the values. For phosphorus, the concentrations of both collection points were equal to 2.0 ± 0.7 mgL^-1^.

### Tensile test, density, empty bed volume and surface area

In Table 1 are the results from tension testing of the coconut husk fibers. Results indicate that the amount of force and maximum tension withheld by the fibers after use in the anaerobic filter is statistically the same as those fibers tested prior to use in the filters. This indicates that the use of coconut husks as filler material within anaerobic filters did not alter the strength of the husks over the two years of study.

**Table 1:** **Mean values of the tensile test**

Parameter	Before using in anaerobic filter	After using in anaerobic filter
Diameter range of ten fibers taken from coconut husks (mm)	8.2 ± 1.8 *a*	8.0 ± 1.1 *a*
Area (mm^2^)	54.2 ± 26.1 *a*	52.0 ± 15.1 *a*
Force (N)	96.7 ± 5.1 *a*	95.7 ± 3.0 *a*
Tension (MPa)	2.1 ± 0.7 *a*	2.0 ± 0.5 *a*

Different lower case letters in each line indicate significant difference (*p < 0.05*).

Table 2 presents the results for density, empty bed volume and surface area determined for the coconut shells. These values are compared to reported values for other commonly used materials.

**Table 2:** **Density, empty bed volume and surface area**

Parameter	Coconut shells		Bamboo rings	Crushed stone	Rasching rings (2 in)*
	Before using in anaerobic filters	After using in anaerobic filters			
Density (kg.m^-3^)	915.9 ± 46.4 *a*	306.3 ± 66.8 *b*	n.a.**	n.a.**	n.a.**
Empty bed volume (%)	62.5 ± 2.4 *a*	81.3 ± 2.7 *b*	75	55	92
Specific surface area (m^2^.m^-3^)	89.6 ± 13.5 *a*	100.3 ± 14.8 *b*	91.8	60.1	103.0

*Henley and Seader (2005). **n.a.: not found in the literature. Different lower case letters in each line indicate significant difference (*p < 0.05*).

We can note that, after the period of residence inside the reactor, there was a significant increase in the empty bed volume, with a change from 62.5 ± 2.4 to 81.3 ± 2.7%. This may be due to the consumption of easily degradable compounds present in the coconut shell. The portion of the husk consisting of fibers has a degradation hampered by the existence of cellulose and woody material, which confers high stability to the biological action (Ohmiya et al. 2005). The properties detailed in Tables 1 and 2 were also assessed every four months (data not shown). At four months of operation, there was no significant difference in the properties evaluated (Table 2), indicating that the coconut husks retain their integrity as a filler medium for long periods of use.

Similar to the increase in empty bed volume over the full operation time, there was an increase in surface area, from 89.6 ± 5.13 to 100.3 ± 14.8 m^2^.m^-3^. This provides the appropriate conditions for the adhesion of a large amount of biomass (Chernicharo 2007). The decrease in density, from 915.9 ± 46.4 to 306.3 ± 66.8 kg.m^-3^, means that it is not necessary to construct a reactor with heavy and expensive structures to withstand increased weight over time.

Regarding the empty bed volume and surface area, it is important to note that, after the residence inside the reactor, results showed values higher than those found in the literature for crushed stone and bamboo rings and similar to the characteristics found for the Rasching rings, filling commonly used in the chemical industry. This can be important in reducing the possibility of clogging of the bed due to the obstruction of the interstices, as explained by Chernicharo (2007).

During the full two years of experimentation, there was never any observable clogging of the system, demonstrating that the use of coconut shell does not create major obstacles to the flow of sewage into the reactor. As the coconut husk has a diameter of about 0.150 m, each of the four parts used in filling the reactor has an average width of 0.018 m and is not so small to cause clogging of the reactor.

Additionally, there was never a need to performed sludge removal, demonstrating that this system requires a low frequency of maintenance. Still, the concentration of suspended solids in the effluent from the reactor remained consistently low, with an average 33 ± 14 mgL^-1^. Dividing the study period into six groups of four months shows that the averages for TSS concentrations were not significantly different (20, 40, 35, 30, 28 and 30 mgL^-1^), indicating the filters did not develop strong drag sludge particles even in the final stage of the study. All concentrations were below the maximum allowable limit for release into bodies of water appointed by the laws of the Brazilian State of Minas Gerais (COPAM 2008), which is 100 mgO_2_L^-1^. Minas Gerais is one of the few states of the Brazilian federation that has specified this parameter in their environmental legislation.

## Conclusions

With the results obtained, it was found that it is possible to employ coconut shells as filler material in anaerobic filters, as they have high resistance to biological degradation. In addition to this, the coconut husks displayed more robust values of empty bed volume and surface area than other low cost materials typically employed.

The anaerobic filter with coconut shells as filling led to a treatment efficiency for COD and BOD compatible with those found in the literature. Finally, the system is simple in terms of operation and maintenance. Therefore, this research has shown that for the construction of anaerobic filters in small communities, especially in developing countries.
